# Rapid response to selpercatinib in RET fusion positive pancreatic neuroendocrine carcinoma confirmed by smartwatch

**DOI:** 10.1038/s41698-024-00659-x

**Published:** 2024-07-31

**Authors:** Barbara Deschler-Baier, Markus Krebs, Matthias Kroiss, Manik Chatterjee, Daniel Gundel, Christian Kestler, Alexander Kerscher, Volker Kunzmann, Silke Appenzeller, Katja Maurus, Andreas Rosenwald, Ralf Bargou, Elena Gerhard-Hartmann, Vivek Venkataramani

**Affiliations:** 1 Comprehensive Cancer Center Mainfranken, University Hospital Würzburg, 97080 Würzburg, Germany; 2 Department of Urology and Pediatric Urology, University Hospital Würzburg, 97080 Würzburg, Germany; 3 Department of Internal Medicine I, Division of Endocrinology and Diabetes, University Hospital, University of Würzburg, 97080 Würzburg, Germany; 4 Department of Internal Medicine IV, LMU University Hospital, Ludwig-Maximilians-Universität München, 80366 Munich, Germany; 5 Hämatologisch-Onkologische Schwerpunktpraxis Würzburg, 97080 Würzburg, Germany; 6 Institute for Diagnostic and Interventional Radiology, University Hospital Würzburg, 97080 Würzburg, Germany; 7 Department of Internal Medicine II, Medical Oncology, University Hospital Würzburg, 97080 Würzburg, Germany; 8 Institute of Pathology, University of Würzburg, 97080 Würzburg, Germany; 9 Bavarian Cancer Research Center (BZKF), 97080 Würzburg, Germany

**Keywords:** Biomarkers, Outcomes research

## Abstract

This case report describes the efficacy of selpercatinib, a selective RET inhibitor, in an unusual case of large-cell neuroendocrine pancreatic carcinoma (LCNEPAC) harboring a *CCDC6*::*RET* fusion. A 56-year-old male with a history of multiple lines of systemic therapies exhibited marked clinical amelioration shortly after initiating selpercatinib within the LOXO-RET-17001 study (ClinicalTrials.gov ID: NCT03157128, first posted: 2017-05-17). Data from the patient’s smartwatch suggested early efficacy before conventional methods, such as serum tumor markers and CT imaging confirmed the antitumor activity. This case not only underscores the efficacy of selpercatinib in treating *RET* fusion-positive rare tumors but also highlights the potential of wearable technology in cancer care. In conclusion, the standard readings from commercially available wearable devices can be useful for the monitoring of treatment response to targeted therapy and may serve as digital biomarkers in clinical trials. This approach marks a significant advancement in patient-centric healthcare, leveraging technology to enhance the effectiveness and precision of treatment evaluation.

## Introduction

Precision oncology has emerged as a pivotal force towards individualized therapeutic strategies. One drug prominently representing this shift is selpercatinib, a selective tyrosine kinase inhibitor, engineered to target aberrations in the *RET* (Rearranged during Transfection) proto-oncogene. *RET* fusions, characterized by the loss of the transmembrane domain, initiate a cascade of oncogenic signals within the cytoplasm by activating the RET kinase domain. This results in the activation of pathways such as PI3K/Akt and RAS/RAF/MEK/ERK, fueling the proliferation and survival of cancer cells. Selpercatinib was engineered to inhibit RET kinase activity specifically by binding to its ATP-binding pocket. This action prevents the kinase from phosphorylating substrates, thereby halting oncogenic signaling^1,2^. The LIBRETTO-001 phase 1/2 trial (NCT03157128) was instrumental in unveiling the efficacy of selpercatinib in managing RET-altered non-small cell lung cancer (NSCLC) and medullary thyroid cancer (MTC), paving the way for its initial regulatory approvals^1,2^. Building on these results, two phase 3 trials further validated selpercatinib’s clinical benefits: The LIBRETTO-431 trial (NCT04194944) established selpercatinib as a frontline therapy for RET fusion-positive NSCLC, while the LIBRETTO-531 trial (NCT04211337), the first randomized study to compare selpercatinib with multikinase inhibitors, reinforced its efficacy in treating RET-mutated MTC^3,4^. Moreover, comprehensive data from the LIBRETTO-001 trial have led to its groundbreaking tissue-agnostic FDA approval for RET-fusion-positive cancers, underscoring the impact of selpercatinib in the field of precision oncology^5,6^. Recently, the European Medicines Agency (EMA) granted approval for selpercatinib in RET-fusion-positive cancers.

This case report describes a patient with large-cell neuroendocrine pancreatic carcinoma (LCNEPAC), exhibiting metastatic dissemination to lymphatic and hepatic sites. The identification of a *CCDC6::RET* fusion prompted inclusion into the LIBRETTO-001 trial. The patient showed a remarkable response to selpercatinib, as evidenced by CT staging and serum tumor markers. Beyond established clinical staging measures, smartwatch-derived data revealed immediate improvements in physical activity (increase in the number of daily steps) and physiological metrics (decrease in baseline heart rate). This case exemplifies the integration of innovative wearable technology into routine cancer care, thus enhancing the granularity of patient-centric monitoring and care.

## Results

### Patient history, re-biopsy, and molecular analysis

**Fig. 1:**
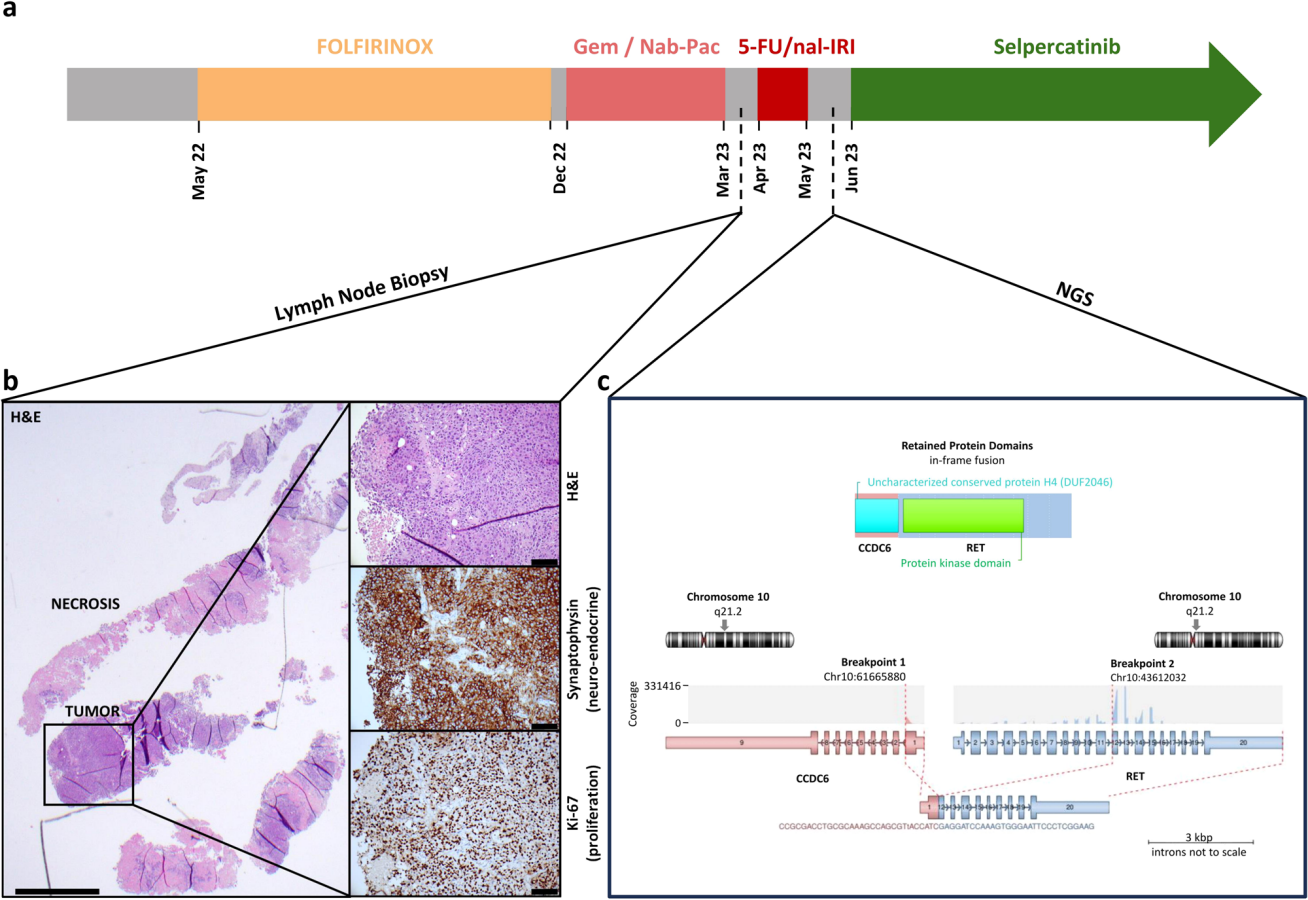
Clinical course, pathology, and genetic fusion in LCNEPAC. **a** Timeline of clinical courses, including treatment duration. **b** Pathologic examination revealed a partially necrotic, poorly differentiated carcinoma that grew in solid sheets or trabeculae without obvious gland formation, morphologically compatible with large-cell neuroendocrine carcinoma. Immunohistochemistry (IHC) showed strong and uniform expression of synaptophysin, along with a high Ki-67 proliferative index of 70–80%. Scale bar (overview): 1 mm; Scale bar (inserts): 100 µm. **c** A schematic representation of the in-frame *CCDC6*::*RET* fusion gene, with the breakpoint indicated on chromosome 10, and the resulting retained protein domains from both CCDC6 and RET. The CCDC6::RET fusion was visualized using Arriba, version v2.4.0^18^ (image modified).

In April 2022, a 56-year-old male received the diagnosis of pancreatic adenocarcinoma with lymph node and liver metastases. The patient first received the FOLFIRINOX chemotherapy regimen (folinic acid, fluorouracil, irinotecan, and oxaliplatin). Subsequent treatment with nab-paclitaxel and gemcitabine led to a mixed response: while liver metastases decreased in size, abdominal and cervical lymph nodes significantly increased in size, most notably the Virchow’s lymph node (located above the left clavicle). Additionally, the patient presented with several cutaneous soft tissue metastases, including a particularly noteworthy lesion adjacent to the right parasternal area. Radiological evaluation revealed extensive retroperitoneal lymph node metastases and an additional lesion in the left adrenal gland. The mixed response indicated the need for a reevaluation of the treatment strategy. Consequently, the patient’s oncologist pursued a re-biopsy of Virchow’s lymph node and changed therapy towards fluorouracil and nanoliposomal irinotecan (5-FU/nal-IRI) (Fig. 1a). Collaborating with the molecular tumor board of the Comprehensive Cancer Center Mainfranken (CCC MF), which has a special focus on community outreach and clinical trial inclusion^7^, the oncologist initiated an extensive histopathological and molecular examination. Of note, the histopathological analysis of the lymph node biopsy revealed a transition from pancreatic adenocarcinoma to large-cell neuroendocrine pancreatic carcinoma (LCNEPAC). This change was supported by the immunohistochemical expression of synaptophysin and Insulinoma-associated protein 1 (INSM1), along with pancytokeratin and cytokeratin 7, and a high Ki67 index. Notably, TTF1 was negative (Fig. 1b and data not shown). The diagnostic shift from pancreatic adenocarcinoma to LCNEPAC may suggest the initial presence of a mixed neuroendocrine non-neuroendocrine neoplasm (MiNEN) subset initially not represented in the pathological specimen. Subsequent genomic profiling, utilizing DNA-based cancer panel sequencing (Oncomine Comprehensive Assay Plus), revealed a homozygous deletion of the *CDKN2A* and *MTAP* gene locus but did not identify further actionable mutations. However, a targeted RNA-based fusion analysis employing the Archer Lung Panel revealed the presence of a *CCDC6::RET* fusion, shown in Fig. 1c. This pivotal finding prompted the molecular tumor board to recommend the patient’s inclusion in the LIBRETTO-001 trial (NCT03157128)^5,6^. Within two weeks after confirming the *RET* alteration and following the ineffectiveness of 5-FU/nal-IRI, the patient was enrolled in the study.

### Robust patient-identified, radiographic, and biomarker responses to Selpercatinib

The initiation of selpercatinib therapy (160 mg twice daily) marked a turning point in the patient’s clinical course. Within a few days, the patient reported pronounced shrinkage of the palpable supraclavicular lymph node and other palpable soft tissue metastases, along with a marked reduction in pain and decreased requirement for hydromorphone. Most remarkably, within 5 days of commencing selpercatinib therapy, the patient’s mobility improved to the extent that a walker was no longer necessary.

**Fig. 2:**
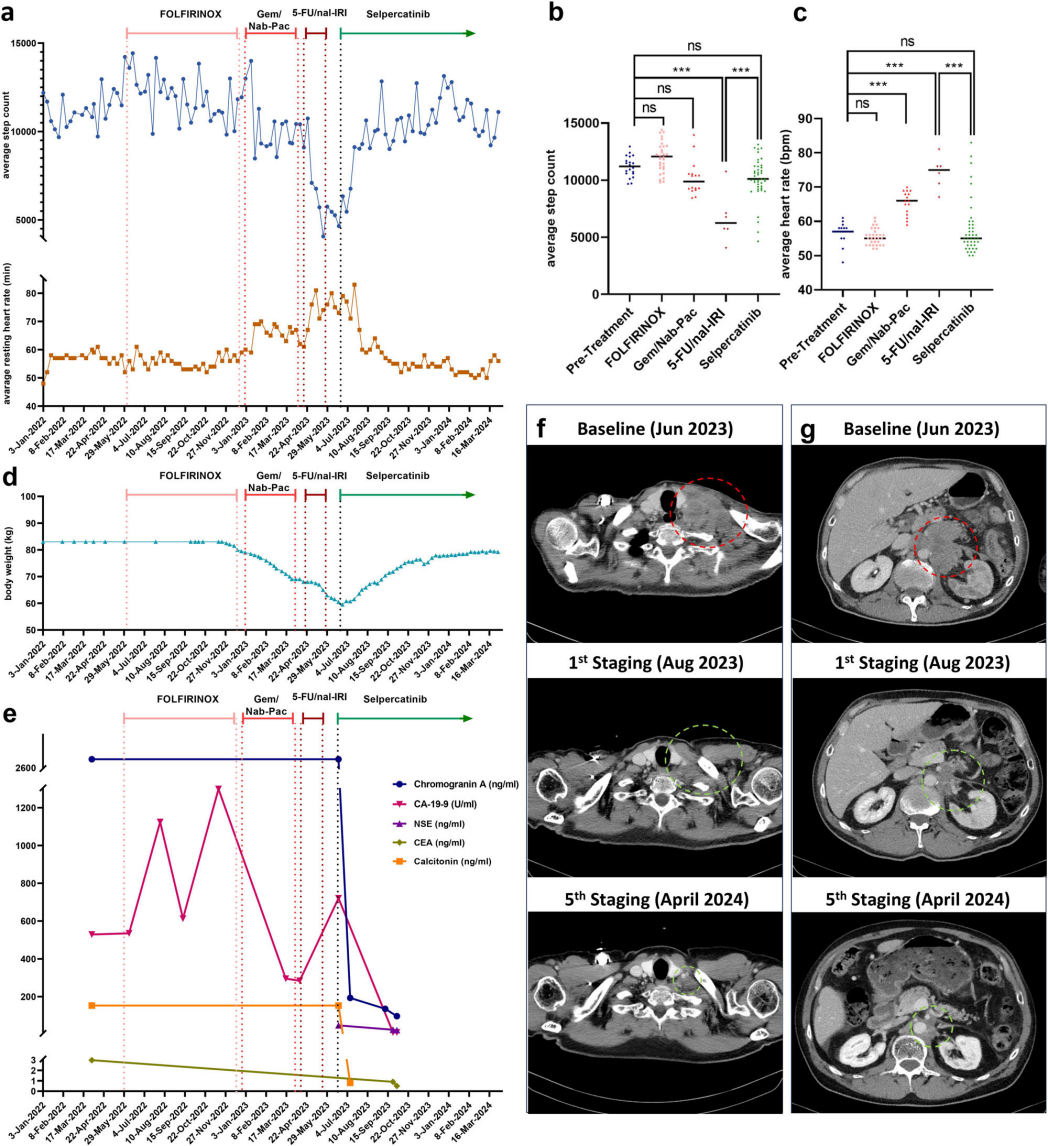
Multimodal assessment of response to selpercatinib. **a** Timeline displaying the patient’s clinical trajectory, with corresponding smartwatch data capturing the variations in average daily step counts (blue line) and heart rates (brown line). Vertical lines mark key interventions in the treatment regimen. **b** Scatter plot demonstrating the statistical analysis of average daily step count and (**c**) average heart rate data in beats per minute (bpm). Analysis of variance (ANOVA) with Bonferroni posthoc analysis was performed to assess statistical differences between phases. Significance is denoted as ns (not significant), ***p < 0.001. **d** Patient’s self-reported body weight in kilograms (kg), measured daily starting from November 14, 2022. Measurements taken before this date were captured at irregular intervals. **e** Changes in serum tumor markers chromogranin A (blue), CA-19-9 (magenta), NSE (purple), calcitonin (orange) and CEA (green), aligned with respective treatment intervals. **f**, **g** Axial CT images of the thorax and abdomen. Pre-treatment images identify metastatic lymph nodes (marked by red dashed circles) in the (**f**) cervical left supraclavicular region and (**g**) retroperitoneum. Post-treatment scans demonstrate significant shrinkage in metastatic burden (green dashed circles), indicative of a robust response to selpercatinib. Although not shown here, the patient additionally demonstrated a significant regression in metastases across various regions. This includes the regression of extensive metastases in the cervical, axillary, and infraclavicular lymph nodes, as well as in the mediastinum and mesenteric regions. Metastases in the retroperitoneal area, contiguous with the primary pancreatic tumor, and multiple soft tissue metastases within the subcutaneous fat of the peritoneum and retroperitoneum also showed notable reduction. Furthermore, increased sclerosis in osseous structures, especially pronounced in the sternum’s body, indicated previously occult disseminated bone metastases.

Of note, the patient wore his smartwatch throughout his complete cancer therapy. Measuring step counts and heart rate offered real-time insights into the patient’s activity levels and physiological responses. As illustrated in Fig. 2a–c, initial chemotherapy phases showed fluctuating step counts, with a downward trend indicating diminished activity, possibly due to adverse effects and disease progression. Each therapeutic switch, from FOLFIRINOX to nab-paclitaxel and gemcitabine, and later to 5-FU/nal-IRI, corresponded with a decrease in step count, reflecting the accumulating impact of the disease and treatment side effects. Conversely, the introduction of selpercatinib marked a positive shift, with a noticeable and rapid increase in step count suggestive of improved mobility and therapeutic response. Concurrently, a significant decrease in average resting heart rate suggested reduced systemic stress and improved overall health. Notably, the patient received the beta-blocker bisoprolol (2.5 mg) from November 2011 until March 2024, to manage asymptomatic coronary heart disease. Smartwatch-recorded data revealed moments where the patient independently adjusted his opioid dosage, mirroring his perceived pain reduction and overall well-being enhancement. This meticulous smartwatch tracking underscored selpercatinib’s impact on quality of life and validated treatment efficacy through improved physical performance indicators. At the same time, his appetite increased, which played a critical role in reversing cancer-associated cachexia^8^. As a result, he gained 20 kg of weight over a 6-month period, thereby markedly enhancing his overall nutritional status and quality of life (Fig. 2d).

Tumor markers exhibited a dramatic drop within 15 days after selpercatinib initiation; calcitonin levels normalized, and chromogranin A levels plummeted from 2900 to 190 ng/ml. Further affirming treatment effectiveness, CA-19-9, NSE, and CEA levels also declined (Fig. 2e).

Cross sectional imaging revealed a marked regression in metastatic lesions. Specifically, metastases in multiple lymph node regions, including those in the retroperitoneal space adjacent to the primary pancreatic tumor, showed a reduction in size. Additionally, metastases in various soft tissue regions and the adrenal glands diminished, and mesenteric lymph node metastases normalized in size. There was also a noticeable regression in cutaneous metastatic areas. Interestingly, increased sclerosis in skeletal regions such as the sternum suggested a response to initially occult bone metastases (Fig. 2f, g). Currently, radiological response to selpercatinib is still ongoing (13 months post initiation).

## Discussion

Evidence of RET fusions in solid tumors has predominantly been documented in thyroid and lung cancers. However, their occurrence in pancreatic carcinoma remains notably rare. According to a comprehensive next-generation sequencing (NGS) study by Kato et al., which analyzed nearly 5000 patient samples, RET fusions were identified in a variety of cancers but were relatively infrequent in pancreatic adenocarcinoma. Out of 160 pancreatic adenocarcinoma samples examined, only a single case was found to have a RET rearrangement, resulting in a prevalence rate of 0.6%, thus highlighting the rarity of such genetic alterations in pancreatic cancer^9^. Contrastingly, the LIBRETTO-001 trial displayed a higher representation of patients with RET fusion-positive pancreatic adenocarcinoma, accounting for 26.7% (12 out of 45 patients) of the study cohort^5^. This discrepancy underscores the potential clinical significance of identifying RET fusions in pancreatic cancer despite their overall low prevalence and suggests a possible avenue for targeted therapy in this subset of patients.

Our report demonstrates the efficacy of selpercatinib in a patient with *RET* fusion-positive LCNEPAC. This response exemplifies the potential advantage of targeted therapies over traditional chemotherapeutics, with selpercatinib not only conferring clinical benefits but also minimizing adverse effects and improving quality of life. Results demonstrated here align with emerging data on selpercatinib’s efficacy across diverse *RET*-altered tumors, including NSCLC, colorectal cancer, salivary gland cancer, pancreatic adenocarcinoma and glioblastoma, thereby indicating its broad applicability^5,10–12^. Beyond established clinical staging parameters, our case report demonstrates the potential of smartwatch-derived data in tracking the immediate response toward targeted therapy. Our results confirm previous findings on the benefit of using smartphones in oncological assessment—for example, several studies have examined the capability of smartphone accelerometers to accurately estimate step counts. These studies consistently found that smartphone readings, serving as digital biomarkers, were valid and reliable^13^. A feasibility study assessed the effectiveness of providing chemotherapy patients with a smartphone equipped with a pedometer application. Patients received a message if their daily step count declined by more than 15% from the initial baseline. The study demonstrated that this intervention was both feasible and effective in identifying chemotherapy toxicity. Another study involving 62 patients undergoing cancer surgery found that analyzing exertional activity using smartphone accelerometer data was effective in distinguishing between patients who had postoperative complications and those who did not^14^.

This case demonstrates the synergistic value of combining smartwatch-derived data with conventional clinical measures, illustrating the progress in precision oncology. Selpercatinib’s role in treating *RET* fusion-positive pancreatic neuroendocrine carcinoma exemplifies the merging of targeted therapy with digital health tools, signifying a shift toward more adaptive, patient-focused oncological care, and emphasizing the importance of digital biomarkers in monitoring and improving treatment outcomes.

However, our study is not without its limitations. Notably, the occurrence of RET fusions as actionable targets is uncommon, and there is an increasing evidence base pointing to both primary and secondary resistance to RET inhibitors in fusion-positive cancers^15,16^. Additionally, while our study greatly benefitted from the patient’s diligent monitoring of his health data via a smartwatch across his entire treatment journey, it is essential to recognize that our patient´s high levels of commitment and compliance may not be universally representative. Nevertheless, this case report highlights the potential benefit of integrating wearable technology and digital solutions into our cancer care algorithms^17^.

In conclusion, this report underscores the efficacy of selpercatinib for *RET* fusion-positive tumors beyond lung and thyroid cancers, supporting its integration into the treatment paradigm for diverse *RET*-altered malignancies. The rapid clinical and symptomatic improvements observed and supported by smartwatch-derived data within days of initiating therapy highlight the drug’s potent therapeutic activity. The exceptional response to selpercatinib highlights the importance of a comprehensive genomic analysis in treating complex cancers like neuroendocrine pancreatic carcinoma. This case not only contributes to the expanding evidence for the efficacy of RET inhibitors but also calls for continued innovation and research into targeted treatments. Finally, we could show that smartwatch-derived information can be a valuable contribution to established clinical staging parameters—especially in terms of tracking the early onset of therapy response.

## Methods

### Participant

The patient received selpercatinib as part of his participation in the phase I/II clinical trial LOXO-RET-17001 (ClinicalTrials.gov ID: NCT03157128, first posted: 2017-05-17) evaluating orally administered selpercatinib for individuals with advanced solid tumors. This included patients with RET fusion-positive solid tumors, medullary thyroid cancer, and other tumors exhibiting RET activations. Enrollment in the trial was preceded by the collection of written informed consent from the patient. Additionally, written informed consent for the publication of this case report and any accompanying images was obtained. The independent assessment of radiological response to treatment was carried out following the RECIST version 1.1 guidelines by a qualified radiologist (C.K.).

### Activity monitoring using wearable technology

In this case report, the Apple Watch Series 3, acquired by the patient, was implemented as a tool to measure physical activity levels and heart rate during the treatment period. This device is equipped with a motion sensor capable of tracking physical activity. The software automatically analyzed the total steps taken each day. To provide a clearer picture of the patient’s mobility trends amidst the natural daily variations in activity, the median value of these daily averages was computed on a weekly basis. This method ensured a stable and accurate analysis of the patient’s mobility changes throughout the treatment, giving insights into the physical condition and any potential treatment impacts on mobility. Additionally, the heart rate monitoring feature of the smartwatch collected data on the patient’s average resting heart rate. By focusing on periods of inactivity, this measurement provided valuable information on the patient’s cardiovascular health and the indirect impact of treatment on overall fitness.

### Molecular testing

Tumor samples were obtained via CT-guided biopsy performed by a radiologist. Formalin-fixed paraffin-embedded (FFPE) specimens derived from tumor samples were reviewed by a pathologist (E.G-H.) to confirm the histopathological diagnosis and to ensure adequate tumor cellularity for analysis. DNA and RNA were extracted from FFPE tissue using a Maxwell^®^RSC instrument (Promega GmbH, Walldorf, Germany) according to standard protocols. Genomic profiling was performed using DNA-based cancer panel sequencing (Oncomine™ Comprehensive Assay Plus) and the Ion Reporter Software v5.18 (Thermo Fisher Scientific, Waltham, MA, USA). Targeted RNA-based fusion analysis was carried out using the Archer™ FUSIONPlex™ Lung v2 panel and the Archer Analysis Software v6.2.3 (ArcherDX/Integrated DNA Technologies, Boulder, CO, USA). Sequencing was performed using semiconductor sequencing technology (Ion GeneStudio S5, Thermo Fisher).

### Ethics

This study complied with all relevant ethical regulations, including the Declaration of Helsinki. The LOXO-RET-17001 study protocol was approved by the Ethics Committee of the Medical Faculty of the University of Cologne (18-408). Written informed consent was obtained from the patient involved in the study.

### Supplementary information

CONSORT 2010 Checklist

## Data Availability

All relevant data are available from the corresponding author upon reasonable request.
