# Supplementary material for: Rapid response to selpercatinib in RET fusion positive pancreatic neuroendocrine carcinoma confirmed by smartwatch
Source: NPJ Precis Oncol. 2024 Jul 31;8:167. doi: 10.1038/s41698-024-00659-x (PMC11291676; doi:10.1038/s41698-024-00659-x)
Supplement: Supplementary file 1 — CONSORT 2010 Checklist [file 41698_2024_659_MOESM1_ESM.doc]

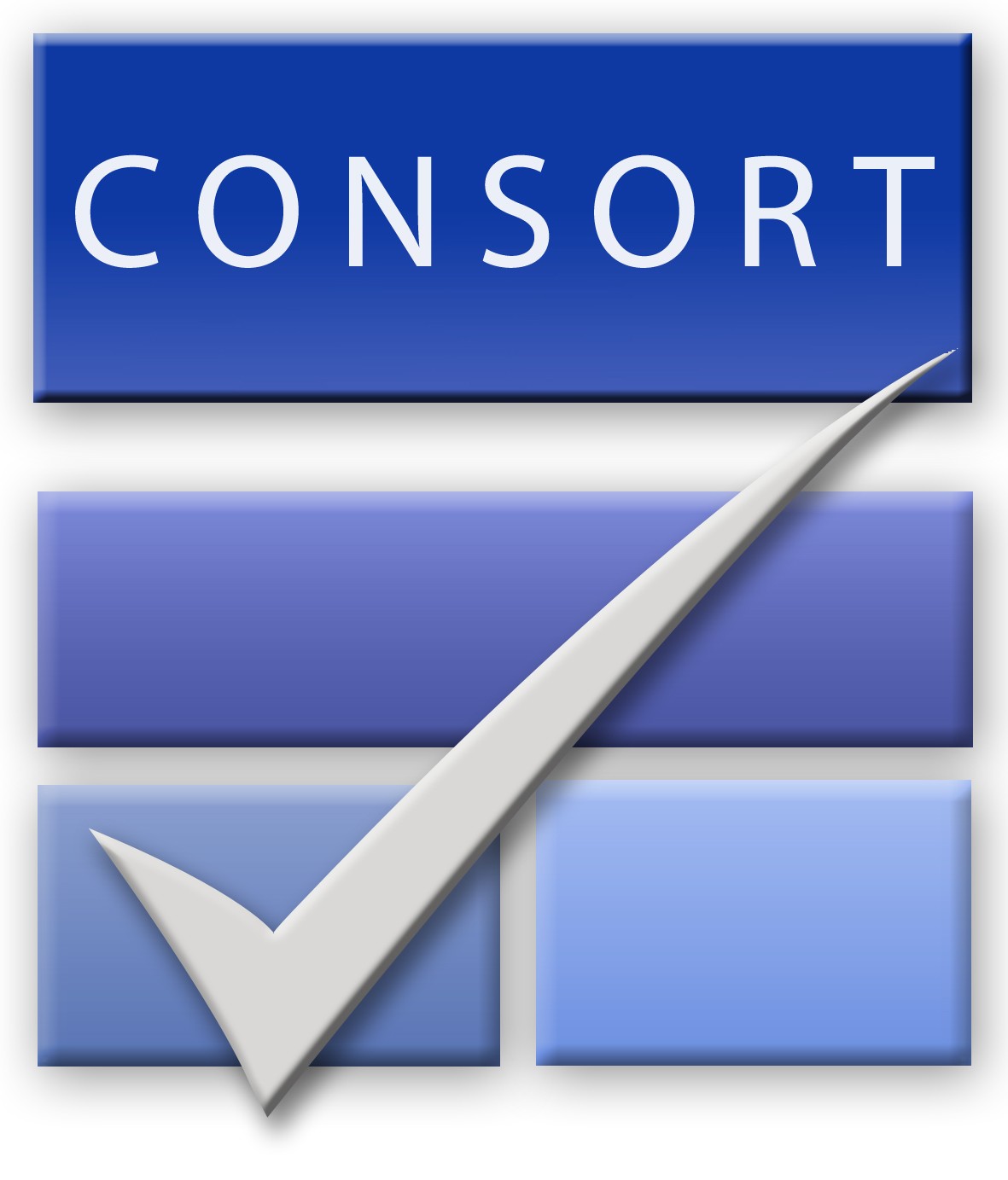
CONSORT 2010 checklist of information to include when reporting a randomised trial*

| Section/Topic | Item No | Checklist item | Reported on page No |
| --- | --- | --- | --- |
| Title and abstract | | | |
|  | 1a | Identification as a randomised trial in the title | N/A |
|  | 1b | Structured summary of trial design, methods, results, and conclusions (for specific guidance see CONSORT for abstracts) | N/A |
| Introduction | | | |
| Background and objectives | 2a | Scientific background and explanation of rationale | page 19 |
|  | 2b | Specific objectives or hypotheses | page 5 and page 26 |
| Methods | | | |
| Trial design | 3a | Description of trial design (such as parallel, factorial) including allocation ratio | pages 5 and page 28 |
|  | 3b | Important changes to methods after trial commencement (such as eligibility criteria), with reasons | Page 2-4 |
| Participants | 4a | Eligibility criteria for participants | Page 6 |
|  | 4b | Settings and locations where the data were collected | Page 7 and 75 |
| Interventions | 5 | The interventions for each group with sufficient details to allow replication, including how and when they were actually administered | Page 47-55  Page 52-53 |
| Outcomes | 6a | Completely defined pre-specified primary and secondary outcome measures, including how and when they were assessed | Page 26, 79 |
|  | 6b | Any changes to trial outcomes after the trial commenced, with reasons | Page 2-3 |
| Sample size | 7a | How sample size was determined | Page 80 |
|  | 7b | When applicable, explanation of any interim analyses and stopping guidelines | Page 82-83 |
| Randomisation: |  |  |  |
| Sequence generation | 8a | Method used to generate the random allocation sequence | Page 85 |
|  | 8b | Type of randomisation; details of any restriction (such as blocking and block size) | N/A |
| Allocation concealment mechanism | 9 | Mechanism used to implement the random allocation sequence (such as sequentially numbered containers), describing any steps taken to conceal the sequence until interventions were assigned | N/A |
| Implementation | 10 | Who generated the random allocation sequence, who enrolled participants, and who assigned participants to interventions | N/A |
| Blinding | 11a | If done, who was blinded after assignment to interventions (for example, participants, care providers, those assessing outcomes) and how | N/A |
|  | 11b | If relevant, description of the similarity of interventions | N/A |
| Statistical methods | 12a | Statistical methods used to compare groups for primary and secondary outcomes | Page 83 |
|  | 12b | Methods for additional analyses, such as subgroup analyses and adjusted analyses | Page 83-83 |
|  | | | |
| Participant flow (a diagram is strongly recommended) | 13a | For each group, the numbers of participants who were randomly assigned, received intended treatment, and were analysed for the primary outcome | Page 82-83 |
|  | 13b | For each group, losses and exclusions after randomisation, together with reasons | Page 55-56 |
| Recruitment | 14a | Dates defining the periods of recruitment and follow-up | Page 56 |
|  | 14b | Why the trial ended or was stopped | Page 56 |
| Baseline data | 15 | A table showing baseline demographic and clinical characteristics for each group | Page 83-84 |
| Numbers analysed | 16 | For each group, number of participants (denominator) included in each analysis and whether the analysis was by original assigned groups | Page 80 |
| Outcomes and estimation | 17a | For each primary and secondary outcome, results for each group, and the estimated effect size and its precision (such as 95% confidence interval) | Page 85-86 |
|  | 17b | For binary outcomes, presentation of both absolute and relative effect sizes is recommended | Page 85 |
| Ancillary analyses | 18 | Results of any other analyses performed, including subgroup analyses and adjusted analyses, distinguishing pre-specified from exploratory | Page 86 |
| Harms | 19 | All important harms or unintended effects in each group (for specific guidance see CONSORT for harms) | Page 88 |
|  | | | |
| Limitations | 20 | Trial limitations, addressing sources of potential bias, imprecision, and, if relevant, multiplicity of analyses | Page 91-92 |
| Generalisability | 21 | Generalisability (external validity, applicability) of the trial findings | Page 85 |
| Interpretation | 22 | Interpretation consistent with results, balancing benefits and harms, and considering other relevant evidence | Page 113 |
| Other information | | |  |
| Registration | 23 | Registration number and name of trial registry | Page 5 |
| Protocol | 24 | Where the full trial protocol can be accessed, if available | Page 97 |
| Funding | 25 | Sources of funding and other support (such as supply of drugs), role of funders | Page 93-97 |

*We strongly recommend reading this statement in conjunction with the CONSORT 2010 Explanation and Elaboration for important clarifications on all the items. If relevant, we also recommend reading CONSORT extensions for cluster randomised trials, non-inferiority and equivalence trials, non-pharmacological treatments, herbal interventions, and pragmatic trials. Additional extensions are forthcoming: for those and for up to date references relevant to this checklist, see [www.consort-statement.org](http://www.consort-statement.org/).
